# Tactile gating in a reaching and grasping task

**DOI:** 10.1002/phy2.267

**Published:** 2014-03-24

**Authors:** Francisco L. Colino, Gavin Buckingham, Darian T. Cheng, Paul van Donkelaar, Gordon Binsted

**Affiliations:** ^1^ School of Health & Exercise Sciences Faculty of Health & Social Development The University of British Columbia Kelowna British Columbia Canada; ^2^ Department of Psychology School of Life Sciences Heriot‐Watt University Edinburgh U.K

**Keywords:** Feed forward, reaching and grasping, tactile gating

## Abstract

A multitude of events bombard our sensory systems at every moment of our lives. Thus, it is important for the sensory cortex to gate unimportant events. Tactile suppression is a well‐known phenomenon defined as a reduced ability to detect tactile events on the skin before and during movement. Previous experiments found detection rates decrease just prior to and during finger abduction, and decrease according to the proximity of the moving effector. This study examined how tactile detection changes during a reach to grasp. Fourteen human participants used their right hand to reach and grasp a cylinder. Tactors were attached to the index finger, the fifth digit, and the forearm of both the right and left arm and vibrated at various epochs relative to a “go” tone. Results showed that detection rates at the forearm decreased *before* movement onset; whereas at the right index finger, right fifth digit and at the left index finger, left fifth digit, and forearm sites did not decrease like in the right forearm. These results indicate that the task affects gating dynamics in a temporally‐ and contextually dependent manner and implies that feed‐forward motor planning processes can modify sensory signals.

## Introduction

In the context of motor output, the sensory system detects, identifies, and recognizes sensory patterns to guide an appropriate response. For example, an actor pours coffee into a mug, but some coffee spills on the side of the mug. Immediately, the actor gets a dish towel and grasps the mug to wipe it clean. Tactile information from the hands becomes particularly important for successfully grasping and cleaning the mug. Logically, it is advantageous for the central nervous system to facilitate processing of signals that convey touch information from the coffee on the mug because decreased friction exists between the surface of the skin and the mug. That is, tactile signals from the fingertips should be readily perceived by the actor, whereas tactile signals that are not relevant to a movement's goal will be ignored.

This reduction in the ability to perceive tactile stimuli during movement is known as “tactile gating” (see Rushton et al. 1981; Chapman et al. 1987). This gating is often measured as the percentage of correct reports of a probe stimulus or measured by calculating sensitivity (*d*'). Several studies (Rushton et al. 1981; Chapman et al. 1987; Milne et al. 1988; Williams et al. 1998; Voss et al. 2008; Buckingham et al. 2010) reported suppression of tactile sensations just before and during movement. The majority of studies (e.g., Chapman et al. 1987; Milne et al. 1988; Williams et al. 1998) have examined sensory suppression in simple motor tasks, such as abducting the index finger. More recently, tactile gating has been examined in a wider variety of visuo‐motor tasks, such as pointing (Buckingham et al. 2010), juggling (Juravle and Spence 2011), grasping (Juravle et al. 2011), and during normal gait (Duysens et al. 1995; Morita et al., 1998; Staines et al. 1998). None of these studies, however, examined how tactile gating manifests at task‐relevant versus task‐irrelevant locations on the moving limb (cf. Williams and Chapman 2002).

Furthermore, these previous experiments have failed to resolve the debate regarding whether the suppression is caused by central or peripheral sources. Indeed, there is evidence that cortical networks involving the prefrontal cortex drive somatosensory gating (Yamaguchi and Knight 1990; Bolton and Staines 2011). Bolton and Staines (2011) observed higher P100 event‐related potential amplitude (ERP), sensitive to the direction of spatial attention (see Hillyard et al. 1998), when tactile stimuli were attended. However, factors such as task difficulty, task type, attentional manipulation, and the characteristics of the stimulus itself can influence how tactile gating manifests. This study directly examined these issues and provides evidence that tactile gating is central in origin, arises from predictive mechanisms (Bays et al. 2006; Voss et al. 2008), and is restricted to task‐specific parts of a moving limb – all within the context of goal‐directed grasping movements.

## Methods

### Participants

Participants (eight women, six men) were recruited from the local graduate and undergraduate population (mean age = 24 years; SD = 3.99). They were all self‐reported right‐handed individuals, had normal or corrected‐to‐normal visual acuity, and reported no previous neurological conditions. Participants gave written informed consent, and all procedures were approved by the local research ethics board.

### Apparatus

An Optotrak Certus (Northern Digital, Inc., Waterloo, Ontario, Canada) tracked at 250 Hz the three‐dimensional position of three infrared‐emitting diodes (IREDs) affixed to the index finger, thumb, and wrist of each participant's right hand. Six custom‐built tactile micromotor vibrators (tactors) were taped to the dorsal surface of the proximal phalanx of the left and right index fingers, the dorsal surface of the proximal phalanx of the fifth finger of both hands, and dorsal surface of the mid‐forearm of both arms. Micromotor vibration stimuli consisted of a single 7.5 msec long vibration burst which caused a 1 mm deformation of the skin resulting in the perception of a readily detectable tap at rest (17 mm long, 7 mm diameter, weight 1 g). Participants were seated in an upright padded chair with the left arm resting on a flat grasping surface that was at the level of the upper abdomen. The right arm always began at the home position that was 35 cm to the right of each participant's midline. The elbow was flexed at 90°.

### Task

On each trial, participants performed speeded reaching and grasping movements to a target object cylinder concluding with a simple lift off the reaching surface. Once the grasping movement was complete, participants made a detection judgment whether a vibration was felt (i.e., yes/no) and where the vibration was felt (e.g., left mid‐forearm, proximal phalanx of the left index finger, proximal phalanx of the left fifth digit, etc.).

Data collection took place inside a small sound‐isolated room. Participants sat in front of the horizontal reaching surface, wearing liquid crystal display goggles to occlude vision during the period between trials. All trials began with the right hand 30 cm to the right of the midline and 15 cm in front of the torso and the left hand in the mirror symmetric location. A computer‐generated tone (2000 Hz, 300 msec duration) warned participants that a trial was imminent and 1 sec later the goggles opened. After a subsequent variable foreperiod (1000–1500 msec) the imperative cue consisting of a piezoelectric auditory buzzer (50 msec duration) was presented and participants reached out and grasped the 2 cm diameter and 5 cm high‐polyvinyl chloride (PVC) cylinder with the index finger and thumb of the right hand. They were required to initiate the movement within 400 msec after the buzzer and complete it in 800 msec or less. Movement initiation was defined as sustained velocity of 50 mm/sec for 50 msec. On each trial, the cylinder was located at one of two possible target locations that the experimenter changed randomly during the intertrial period. The locations were 5 cm to the left or right of a position 25 cm directly anterior to the home location for the right hand to prevent participants from predicting target location.

The micromotor vibrations occurred during one of several epochs relative to the imperative cue from 0 msec (at the same time as the imperative cue) to 360 msec (after the imperative cue) in 60 msec bins (i.e., 0, 60, 120, 180, 240, 300, and 360 msec). Once a trial was successfully completed, the LCD goggles closed and participants made a yes/no choice (Y/N) regarding the occurrence of a vibration. In addition, if a vibration was detected, the participant verbally indicated where on the body the vibration was felt (e.g., “left index finger”). There were 10 trials per epoch per vibrator motor (i.e., 10 trials with a delay of 0 msec, 10 trials with a delay of 60 msec, etc.). In addition there were 10 catch trials per motor in which no vibration was delivered, to assess participants' false alarm rate. Each experimental session, thus, comprise 420 trials and lasted between 100 and 120 min. Participants were given opportunities to rest throughout the procedure.

### Data analysis

All trial data were segmented into 60‐msec time bins to achieve temporal accuracy because a reaction time of any given trial may widely differ. To capture the time at which the stimulus was delivered relative to movement onset, we subtracted each participant's reaction time (for each trial) from the time relative to the imperative cue (e.g., 60–300 msec = −240 msec). Nine time bins were created such that they collectively spanned 359 msec before movement onset through 180 msec after movement onset. The time bins were organized as follows: −359 to −300 msec, −299 to −240 msec, −239 to −180 msec, −179 to −120 msec, −119 to −60 msec, −59 to 0 msec, 1 to 60 msec, 61 to 120 msec, and 121 to 180 msec. Every participant's set of trial data were organized into the stimulation epochs relative to the imperative cue (see above). However, too few cases were included into the first and last time bin (i.e., −359 to −300 msec and 121 to 180 msec, respectively) and, therefore, excluded from further analysis.

Sensitivity (*d*') and criterion (*C*) were calculated for every condition within each participant (Geschieder 1997). Sensitivity was calculated by subtracting the false alarm *z*‐score (*Z*_fa_) from the hits *z*‐score (*Z*_h_; see Geschieder 1997, p. 119). False alarm rates were pooled together across all conditions and were used to calculate *d*'. Half the sum of *Z*_h_ and *Z*_fa_ resulted in *C*. Negative *C* values reflect bias toward frequent “yes” responses, whereas positive values of *C* reflect bias toward frequent “no” responses (Gesheider, 1997). *C* was chosen because the range of *C* does not depend on *d*' (Geschieder 1997).

In addition to the detection variables, several different movement performance variables were also monitored. These included reaction time, movement time, peak velocity, peak acceleration, and peak grip aperture. All detection and movement performance variables were submitted to a 6 (vibration location: left and right index finger, left and right fifth digit, left and right forearm) × 7 vibration (−299 to −240 msec, −239 to −180 msec, −179 to −120 msec, −119 to −60 msec, −59 to 0 msec, 1 to 60 msec, 61 to 120 msec) epoch repeated‐measures analysis of variance (ANOVA_RM_). All statistically significant effects and interactions were subjected to paired sample *t*‐tests for all possible pairwise comparisons with no correction for multiple comparisons. Statistical significance was set to *P *<* *0.05. Gating was defined as a significant reduction in detection and *d*' relative to the first time bin.

### Determining gating onset

In addition to the group average detection data, detection rates over time were calculated and fit with a four‐parameter sigmoid regression curve (SigmaPlot, SYSTAT Software, Inc., San Jose, CA). This was done to determine when gating occurred relative to movement onset. First, participant data were screened to determine whether gating was observed. In the present sample (*n *=* *14), two participants did not show gating. As the purpose of the current work was to examine tactile gating, we focused our analysis on the subset of the sample that did experience tactile gating. In the subset who showed gating, gating onset was determined by calculating the point in time of the greatest slope in the sigmoid regression curve. We examined gating onset relative to reaction time using a group‐wise one‐sample *t*‐test (*α *= 0.05). We also correlated gating onset with reaction time using Pearson's correlation (see Buckingham et al. 2010).

### Baseline detection at rest

To control for any potential differences in tactile sensitivity across the stimulation sites, eight (*n* = 4 women) of the original 14 participants completed a follow‐up baseline condition. In this condition, participants were tested with the same vibrator motors adhered to the same testing sites (i.e., left and right fifth digits, left and right index fingers, left and right forearms) after giving informed consent. The protocol consisted of randomly vibrating one site per trial at one of seven vibrator activation intervals (1, 2, 3, 4, 5, 6, or 7 msec) while both arms remained stationary. The longest vibration duration was similar to that used in the main experiment (i.e., 7.5 msec). Eight repetitions were completed for each combination of stimulation site and duration resulting in 336 stimulation trials. In addition, the same number of trials without stimulation were randomly interspersed throughout the protocol, resulting in a total of 672 trials. At the end of each trial, participants were required to indicate whether they felt the stimulation and, if so, at what site. The statistical design was similar to that performed in the main experiment.

## Results

### Baseline detection at rest

Baseline results demonstrate differences between stimulation sites only at the shortest stimulation time (i.e., 1‐msec stimulation). The interaction between vibration site and vibration duration achieved significance, *F*_30,150_ = 2.45, *P *=* *0.0001. The main effect of vibration site achieved significance (*F*_5,35_ = 4.69, *P *= 0.002), and the main effect of vibration duration achieved significance (*F*_6,42_ = 32.9, *P *=* *0.0001). Subsequent simple main effects analyses were conducted targeting the difference in detection rates across sites within each vibration duration (e.g., detection rates of all vibration sites at the 1‐msec duration, and so forth). At the 1‐msec duration, there was a main effect of vibration site (*F*_5,35_ = 3.39, *P *=* *0.013). Least significant difference (LSD) post hoc comparisons revealed significantly lower detection rates at the left forearm compared to the ipsilateral index finger and fifth digit (*P *=* *0.015 and *P *=* *0.012, respectively). On the right side, post hoc comparisons only revealed a significant difference in detection rate between the right forearm and the right index finger (*P *=* *0.042). Hence, on the right side, the lowest detection rate was observed at the right forearm. Importantly, there were no differences between the index fingers, fifth digits, or forearms (all *P*s > 0.10), indicating equivalent detection rate performance between contralateral sites. There were no significant effects at any of the other durations (all *P*s > 0.15), with the interesting exception at the 6‐msec duration that achieved a main effect of vibration site (*F*_5,35_ = 3.08, *P *=* *0.021). However, LSD post hoc comparison did not reveal any statistically significant differences in detection rate (all *P*s > 0.05) at the 6‐msec duration.

### Sensory detection

**Figure 1:**
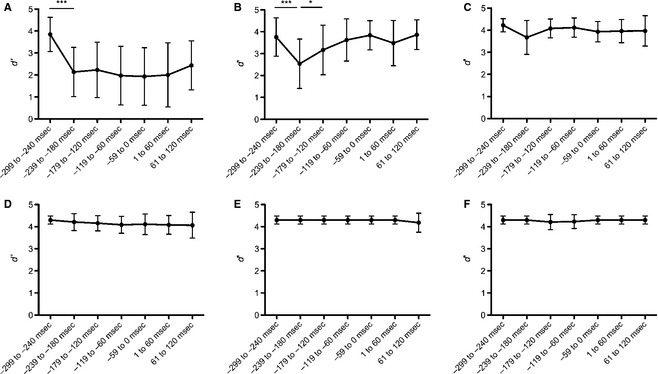
Sensitivity (*d*') calculated from hits and false alarms when the right or left arm was stimulated with vibration at various times relative to movement onset. (A) Right forearm. (B) Right fifth digit. (C) Right second digit. (D) Left forearm. (E) Left fifth digit. (F) Left second digit. For the right arm, *d*' was reduced considerably over the second and third stimulation times and remained diminished; was transiently decreased then returned to baseline at the fifth digit; and remained unchanged at the second digit. For the left arm, *d*' remained constant at all stimulation sites and time. Error bars denote standard deviation. *P <* *0.05. **P <* *0.01.

For gating onset and movement data summaries see Tables 1 and 2. The omnibus repeated‐measure ANOVA of *d*' revealed significant main effects of vibration location, *F*_5,65_ = 24.177, *P *<* *0.001, and vibration epoch, *F*_6,78_ = 11.370, *P *<* *0.001. There was also a significant two‐way interaction between vibration location and vibration epoch (*F*_20,390_ = 6.495, *P *<* *0.001). Subsequent one‐way repeated‐measure ANOVAs were performed to break down the significant two‐way interaction. Post hoc comparisons for vibration location confirmed that the right limb displayed lower sensitivity (left and right fifth digits, *P *=* *0.004; left and right second digits, *P *=* *0.018; left and right forearms, *P *<* *0.0001). A significant effect of vibration epoch was found only at the right fifth digit (*F*_6,78_ = 6.840, *P *<* *0.0001) and at the right forearm (*F*_6,78_ = 11.571, *P *<* *0.0001). There were no significant effects at all other vibration locations (*P*s > 0.09). Post hoc comparisons of the right fifth digit revealed that the −239 to −180 msec time bin decreased the most in sensitivity and it was significantly different from all other time bins (*P*s < 0.05), with the largest difference between that and the preceding time bin (*∆* = 1.22, *P *<* *0.0001). Post hoc comparisons of the right forearm confirmed that largest decrease occurred between the −299 to −240 msec and the −239 to −180 time bin (*∆ *= 1.7, *P *<* *0.0001). There were no significant differences between all other time bins (*P*s > 0.25; see Fig. 1; also see Table 3 for proportion of correctly detected stimuli across time bins).

**Table 1:** Individual participant mean movement onset and tactile gating onset relative to movement onset.

Participant	Mean reaction time, msec (SD)	Gating onset, relative to reaction time; Mean (SD)
1	235 (9)	160 msec before
2	263 (10)	152 msec before
3	275 (14)	208 msec before
4	244 (6)	No gating
5	281 (9)	213 msec before
6	299 (10)	163 msec before
7	321 (14)	212 msec before
8	296 (14)	183 msec before
9	321 (14)	224 msec before
10	258 (7)	159 msec before
11	242 (10)	173 msec before
12	235 (8)	No gating
13	251 (10)	53 msec before
14	295 (12)	222 msec before
Mean	273 (32)	177 (45) msec before

Tactile gating was not observed in participants 4 and 12. Therefore, they did not contribute to the calculation of the mean values at the bottom of the table. Timing values are rounded up to the nearest millisecond.

**Table 2:** Mean movement performance and kinematic data from all participants across all vibration conditions.

Movement parameter	Left fifth digit	Left index finger	Left forearm	Right fifth digit	Right index finger	Right forearm
Reaction time, msec	272 (29)	274 (31)	271 (30)	273 (31)	272 (28)	272 (30)
Movement time, msec	594 (154)	593 (168)	596 (172)	598 (177)	599 (170)	595 (169)
Peak velocity, mm/sec	1225 (102)	1230 (110)	1235 (117)	1222 (104)	1226 (104)	1224 (107)
Peak acceleration, mm/sec^2^	9066 (1481)	9091 (1499)	9161 (1547)	9028 (1467)	9110 (1535)	9142 (1470)
Peak grip aperture, mm	64.9 (0.4)	65.3 (0.4)	64.9 (0.3)	64.5 (0.5)	64.8 (0.2)	64.7 (0.3)

Values are reported mean followed by standard deviation (SD) in parentheses.

**Table 3:** Average proportion of correctly detected stimuli across all participants.

Stimulation times (msec)	L5D	L2D	LF	R5D	R2D	RF
−299 to −240	1.0	1.0	1.0	0.89	0.99	0.93
−239 to −180	1.0	1	0.98	0.63**	0.91	0.52**
−179 to −120	1.0	0.98	0.98	0.77*	0.97	0.54**
−119 to −60	1.0	0.99	0.98	0.87	0.97	0.50***
−59 to 0	1.0	1.0	0.97	0.93	0.96	0.48***
1 to 60	1.0	1.0	0.97	0.84	0.96	0.53***
61 to 120	0.98	1.0	0.96	0.87	0.94	0.59***

The first column shows the time bins in which the detection data were calculated. *P <* *0.05, **P <* *0.01, ***P <* *0.001. Collective false alarm rate was 0.02% across all participants.

**Figure 2:**
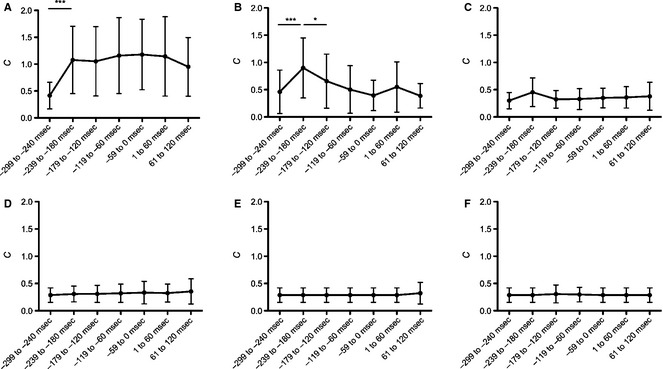
The top panel depicts criterion (*C*) calculated from hits and false alarms when the left and right arms were stimulated with vibration. (A) Right forearm. (B) Right fifth digit. (C) Right second digit. (D) Left forearm. (E) Left fifth digit. (F) Left second digit. *C* plotted on the *y*‐axis. Time relative to movement onset is plotted on the *x*‐axis. Error bars denote standard deviation. *P <* *0.05. **P <* *0.01.

However, *d*' must be measured in light of a criterion measure as it is known that changes in *d*' can simply be due to confounding changes in criterion as opposed to a change in the sensitivity of sensory receptors (Geschieder 1997). Therefore, *C* was calculated for all conditions for every participant. An omnibus repeated‐measure ANOVA was performed to analyze *C* and found the main effects of vibration location (*F*_5,65_ = 22.533, *P *<* *0.0001) and vibration epoch (*F*_6,78_ = 9.041, *P *<* *0.0001). A significant two‐way interaction between vibration location and vibration epoch was found (*F*_30,390_ = 7.068, *P *<* *0.0001). Post hoc comparisons of vibration location confirmed that *C* was higher at the right limb (left vs. right fifth digits, *P *=* *0.006; left vs. right second digits, *P *=* *0.019; left vs. right forearms, *P *<* *0.0001). Subsequent one‐way repeated‐measure ANOVAs were conducted to investigate the vibration epoch revealed significant effects at the right fifth digit (*F*_6,78_ = 7.342, *P *<* *0.0001) and right forearm (*F*_6,78_ = 8.733, *P *<* *0.0001). Post hoc comparisons of the right fifth digit showed the −239 to −280 msec time bin was significantly increased relative to all other time bins (*P*s < 0.05; see Fig. 2). Comparisons of the right forearm revealed there was a significant increase in *C* after the −299 to −240 msec time bin (*∆ *= −0.662, *P *=* *0.001). This increase persisted throughout all time bins (all other *P*s < 0.002).

### Determining gating onset

In addition to the group averaged detection data, individual detection rates from each individual participant's left and right forearm were fit with a four‐parameter sigmoid regression curve. The index finger and fifth digit stimulation sites were not considered as no gating was observed to occur at these sites. These curves highlight the sharp drop in detection rates observed in most participants. This pattern is similar to that observed during single‐joint movements (e.g., Chapman et al. 1987). The time point of the steepest slope (i.e., the highest rate of change) of each curve was calculated and offers a measure of tactile suppression onset. Tactile suppression occurred before movement onset in the vast majority of the sample (see Table 1). A one‐sample *t*‐test was conducted and found that tactile suppression occurred, on average, 177 msec before movement onset, *t*(11) = 6.443, *P *<* *0.0001. Additionally, we noted a significant Pearson's correlation between individual reaction times and suppression onsets, *r*(11) = 0.648, *P *<* *0.05. In particular, participants with longer reaction times also tended to have earlier gating onsets. The present correlation supports the central thesis of this study that sensory gating is unlikely to be a result of sensory reafference.

## Discussion

Previous studies have demonstrated that tactile gating occurs before and during a movement (e.g., Chapman et al. 1987; Milne et al. 1988; Williams et al. 1998). However, they have only examined tactile gating in the context of simple movements in which there would be no reasonable expectation for task‐relevant tactile information. By studying tactile gating within the context of goal‐directed movement we can deconstruct the influence of task on tactile gating. This study aimed to examine the above issues and hypothesized that tactile gating has a central in origin, arises from predictive mechanisms (Bays et al. 2006; Voss et al. 2008), and is restricted to task‐specific parts of a moving limb. Indeed, the present data support the hypothesis that tactile information is attenuated prior to and at the start of a reach and grasping movement and likely before onset of muscle activity (see Buckingham et al. 2010; Cavanagh and Komi 2006). Interestingly, gating was observed at the right forearm (i.e., at the limb that made the reaching and grasping movement).

### Baseline detection at rest

Baseline data clearly show that there was no reduction in detection rates across most stimulus durations, except for the 1‐msec stimulus duration. Also, there were no detection rate differences between stimulation sites at most stimulus durations (except at the 1‐msec duration). The stimulus duration used in the main grasping experiment corresponded to the longest stimulus duration in the baseline study. More importantly, there were no differences across appendages (i.e., no difference between index fingers, fifth digits, and forearms). In light of this baseline data, it is unlikely that detection results from the grasping study are the result of solely baseline detection differences between stimulation sites.

### Detection data

The detection data demonstrate that the tactile gating pattern is affected by task demands. That is, in the right arm substantial suppression occurred in the forearm; whereas it was only transiently present in the fifth digit and did not occur at all in the index finger. By contrast, there was no suppression across all three stimulation locations in the stationary left limb (see Fig. 1).

These data are the first to provide evidence for the existence of a relationship between tactile suppression within the moving limb and the task that limb must perform. In particular, we have demonstrated that the presence of tactile suppression depends on the limb being moved, the segment that contacts an object, and when it makes contact. Traditionally, tactile suppression at a moved limb was observed to be strongest at the limb segment that moved. For example, Chapman et al. (1987) observed tactile suppression to be maximal at the movement effector (in that case the index finger during finger abduction) and systematically reduced the farther away the segment was from the effector. However, in finger abduction there is no expectation for tactile information to be relevant for successful goal completion and, therefore, no need for it to remain effective. By contrast, subcutaneous afferents in the index finger play a crucial role in detecting excessive or insufficient fingertip forces (Johansson and Flanagan 2009) when performing the reach to grasp movements examined in the current work. Thus, the lack of suppression that we observed for the right index finger reflects the context‐dependent requirement for the afferents to maintain their sensitivity.

The findings from the current experiment clearly demonstrate that tactile gating is more complex than had previously been reported. Sensitivity (*d*') calculations show a reduced sensitivity in the moved limbs that are confined to the forearm of the moving limb but not the index finger or the stationary limb. Importantly, *d*' was found to be reduced throughout the course of the movement for the forearm of the moving limb only.

In addition to this contextually specific nature of the suppression, there was also evidence of temporal specificity. In particular, the majority of participants experienced tactile suppression before movement onset. This premovement gating coupled with an increased criterion suggests that the suppression stems from a centrally generated predictive sensorimotor planning mechanism. This is further supported by the fact that there was a clear relationship between individual reaction times and suppression onsets (Buckingham et al. 2010). Taken together, this evidence implies that tactile suppression is a consequence of movement preparation – an event that clearly works centrally and before movement takes place.

### Importance of task

In contrast to previous research on tactile gating, the current work has shown that task demands play a significant role in the modulation of tactile gating, with increasingly lower levels of tactile gating at areas closer to the limb segment that contacts the target surface. For the first time, we have shown that tactile suppression is not observed at points of contact with an object in a task requiring tactile feedback. The observation of tactile gating before movement onset suggests that central mechanisms preemptively change the ability to detect tactile events according to the likelihood that a specific limb segment will receive tactile information during the course of a movement. This mechanism is consistent with a feed‐forward mechanism that specifies the expected sensory dynamics throughout a movement.

Despite these striking data, other alternative explanations need to be considered before making a conclusion. The present results are difficult to reconcile with the well‐documented effect of tactile gating while passively moving a limb (Williams and Chapman 2002). In the Williams and Chapman's (2002) study, a predictive mechanism is unlikely to account for the gating effect since tactile gating occurred in the absence of active movement. In other words, there could not be a central motor command in this context and, by extension, no predictive sensorimotor planning signal. In the case of gating without a motor command, a “postdictive” explanation would suggest gating occurs as a result of sensory inflow in the presence of other sensory events (also see Chapman and Beauchamp 2006 for a demonstration of the effect of task on tactile gating). Present accounts of the pain gating mechanism agree with the postdictive explanation, the best known example being the inhibitory inputs from large (Aβ) fibers to the dorsal horn of the spinal cord (Melzack and Wall 1965). However, the present data demonstrate the occurrence of gating *before* movement opposes the postdictive view. Grasping is a complex movement that requires relevant sources of tactile information. However, in simple single‐joint movements, the central nervous system would not predict that tactile information would be used later in the movement and, therefore would be more likely to gate that information. By contrast, tactile gating would not occur at the specific effectors in a grasp (i.e., the fingers and thumb) because tactile information will be a relevant source of information.

### Mechanisms underlying tactile gating

Functional magnetic resonance imaging (fMRI) has shed some light on the neural mechanisms underlying somatosensory gating effect. Indeed, decreased blood–oxygen‐level dependent (BOLD) signal relative to baseline was observed at the parietal operculum when tactile gating was induced (Jackson et al. 2011) and this reduction was only observed during movement preparation. However, Jackson et al. (2011) raise important questions regarding the mechanisms of somatosensory gating.

There are two seemingly complementary arguments. One side argues that somatosensation should be enhanced if an effector will be at close proximity to a target, as predicted by the premotor theory of attention (Rizzolatti et al. 1987). This idea underlies observations of enhanced somatosensation at an effector when that effector is in close proximity to the target (Huttunen et al. 1996). This idea lends credence to claims of “active” touch during exploratory hand movements.

Conversely, feed‐forward model accounts argue that self‐produced sensory events convey little novel information and should be, therefore, attenuated. This is critical because external events carry important information that may be crucial to an organism's survival (Sperry 1950; Von Holst and Mittelstaedt 1954; Bell 2001). Forward models are proposed to generate estimates of sensory consequences of movements and cancel those afferent signals that match the signals predicted by the forward model. Therefore, resources may be dedicated to the preferential processing of externally generated events (Blakemore et al. 1998; Frith et al. 2000; Voss et al. 2006). A recent study (Parkinson et al. 2011) reports data that were consistent with our contention that movement planning attenuates tactile perception. Parkinson et al. (2011) had participants make reaching movements in response to a visual cue and provided tactile stimuli at various points before or after movement onset. The authors predicted, and demonstrated, that a tactile stimulus would need to be delivered to the moving limb at an earlier point in time for participants to judge movement onset and tactile stimulus delivery occurring simultaneously (Parkinson et al. 2011); this finding suggests motor planning leads to tactile gating at the limb that is about to move. Furthermore, these authors also demonstrated that the parietal operculum (secondary somatosensory cortex, S2) was found to express less BOLD response when tactile stimulation occurred at the moving arm compared to the BOLD when the limb was stationary.

It is possible that prefrontal cortex (PFC) provided the inhibitory input to somatosensory cortex in this study. Indeed, PFC is known to exercise inhibitory control over incoming somatosensory input (Yamaguchi and Knight 1990). Yamaguchi and Knight (1990) observed enhanced early sensory‐evoked potentials (SEP) in the patients with PFC damage compared with control participants. However, it remains unclear how does PFC function when tactile input is relevant to the goal of a task. Chapman (1994) argues that tactile gating is largely central in origin for two reasons. First, gating often occurs before EMG activity in the limb that will move; second, peripheral reafference does not have any effect on evoked potentials due to peripheral stimulation. Likewise, the present experiment shows tactile gating to occur before movement onset. Therefore, it is unlikely that peripheral reafference plays a role in tactile gating. Additionally, pre movement gating has been demonstrated to occur in primate spinal cord via presynaptic inhibition of sensory inputs (Seki et al. 2003). However, the present experiment does not show the time course of tactile gating throughout the grasping movement.

It is, however, possible that participants might have experienced prolonged tactile gating after movement onset. Indeed, previous studies observed reduced H‐reflex amplitude during passive lower limb movements (Brooke 2004) and reduced SEP amplitude for passive upper limb movements (Jones 1981; Rushton et al. 1981). SEP responses from passive limb movements may reflect sensory reafference to spinal cord sensory neurons via inhibitory interneurons and, subsequent presynaptic inhibition of the same sensory neurons in the spinal cord. Thus, any ascending sensory volleys are effectively prevented from reaching higher centers (Brooke 2004). However, there are also central signals from the brain that show sensory attenuation in response to motor commands (Shimazu et al. 1999; Ogata et al. 2009) and that these signals modulate sensory input based on the task relevance (Staines et al. 2000). Indeed, somatosensory evoked potentials are attenuated in response to descending motor commands.

It is also well‐known that somatosensory evoked potentials are reduced before and during movement. SEPs provide clues regarding the mechanisms underlying sensory gating and have shed light on the influence of movement itself and highlight the importance of task. In a study by Shimazu et al. (1999), gating was induced in response to a simple finger and wrist extension and frontal N30, parietal P30, and central N60 SEPs were reduced relative to when movements were not performed. Importantly, Shimazu and colleagues observed that the P14 subcortical potential, the N20 from the primary sensory cortex, and the frontal P22 generated from motor cortex were unchanged. The authors concluded that SEP gating was unaffected by muscle afferent signals. However, it is unclear whether tactile gating occurs in response to the motor command itself. Ogata et al. (2009) provide a clue to this possibility. Ogata and colleagues asked participants to make self‐initiated movements. Most studies (this study included) elicit motor output in response to an imperative cue. By contrast, having participants generate self‐initiated movements has the advantage of allowing an investigator to record movement‐related cortical potentials (MRCP) such as the Bereitschaftspotential (BP) that precedes movement onset. Ogata et al. (2009) demonstrated that the P27 potential recorded at C3′ (2 cm posterior to C3) was found to be different from the resting baseline during the 1500 msec premovement time epoch. Other sensory potentials progressively became reduced as movement initiation approached, with most potentials significantly reduced approximately 500 msec before movement initiation. The SEP reduction time course closely resembles the BP time course and these processes may be correlated (Ogata et al. 2009). Furthermore, evidence indicates that BPs are generated in the supplementary motor area (Neshige et al. 1988).

Given this context, we suggest that the present results might reflect efference copy signals originating from the motor cortices affecting neuronal activity in the primary sensory cortex, thus, gating task‐irrelevant somatosensory signals. Unfortunately, Ogata and colleagues did not measure the correlation between SEP gating and BP generation. Therefore, future research should investigate the possibility of this correlation. Surely, this solidifies the link between motor planning and sensory function. Indeed, sensory gating is certainly influence by task and the expectation of receiving sensory feedback (see above). Staines et al. (2000) tested whether SEPs are influenced by task. They chose to stimulate either the tibial nerve or the sural nerve, testing proprioceptive and cutaneous inflow, respectively. They also presented cutaneous stimuli in the absence of movement and tested proprioceptive function by asking participants to match the passively moved left foot with the right foot. Their principal finding was that SEP gating was modulated by the task demands. Specifically, SEPs generated during the passive movement and cutaneous conditions were suppressed when the sural nerve was stimulated, leaving the SEP generated during the position matching condition relatively unmodulated. Conversely, when the tibial nerve was stimulated SEPs generated in the cutaneous condition were reduced with passive movement and position matching. Staines and colleagues support the position that sensory input can be affected at early stages of processing and that sensory gating is sensitive to task demands. The present results support this position, as tactile stimuli delivered to the right forearm were gated but tactile inputs from the right index finger and fifth digit were unaffected. However, again, the task employed by Staines and colleagues was a passive movement of the left foot. Therefore, there would not have been a motor plan generated to elicit gating. This difference in task makes comparison with this study somewhat troublesome because gating in this study and gating observed by Staines and colleagues must have been generated by different sources.

In the present experiment, we observed sensory attenuation (or, gating) at specific regions of the moving effector. That observation lends support to the feed‐forward model argument that irrelevant sensory events become attenuated if those sensory events do not convey any novel information. However, no sensory attenuation was observed at the location of the right second digit (i.e., index finger), giving the impression that information from the index finger would provide useful information for the purpose of grasping an object. Future studies will be directed at further disentangling the present observation and answering whether the central mechanism attenuates afferent signals deemed irrelevant or optimize inflow by facilitation of afferents from regions that come into contact with objects.

## Conclusions

The current data provide new insights into how the largely understudied phenomenon of tactile gating occurs in the context of movement planning. Based on the fact that it was observed before movement onset, our results are consistent with the fact that tactile gating is a centrally driven effect. Furthermore, tactile gating was not observed to be a global effect across both limbs. Rather, it appears to be specific to the to‐be moved effector and specific to segments of skin in that moved effector. Central mechanisms are able to modulate tactile gating depending on the predicted relevance of tactile information. This observation shows that feed‐forward mechanisms modulate sensorimotor networks, likely optimizing sensory input.

## Conflict of Interest

None declared.
